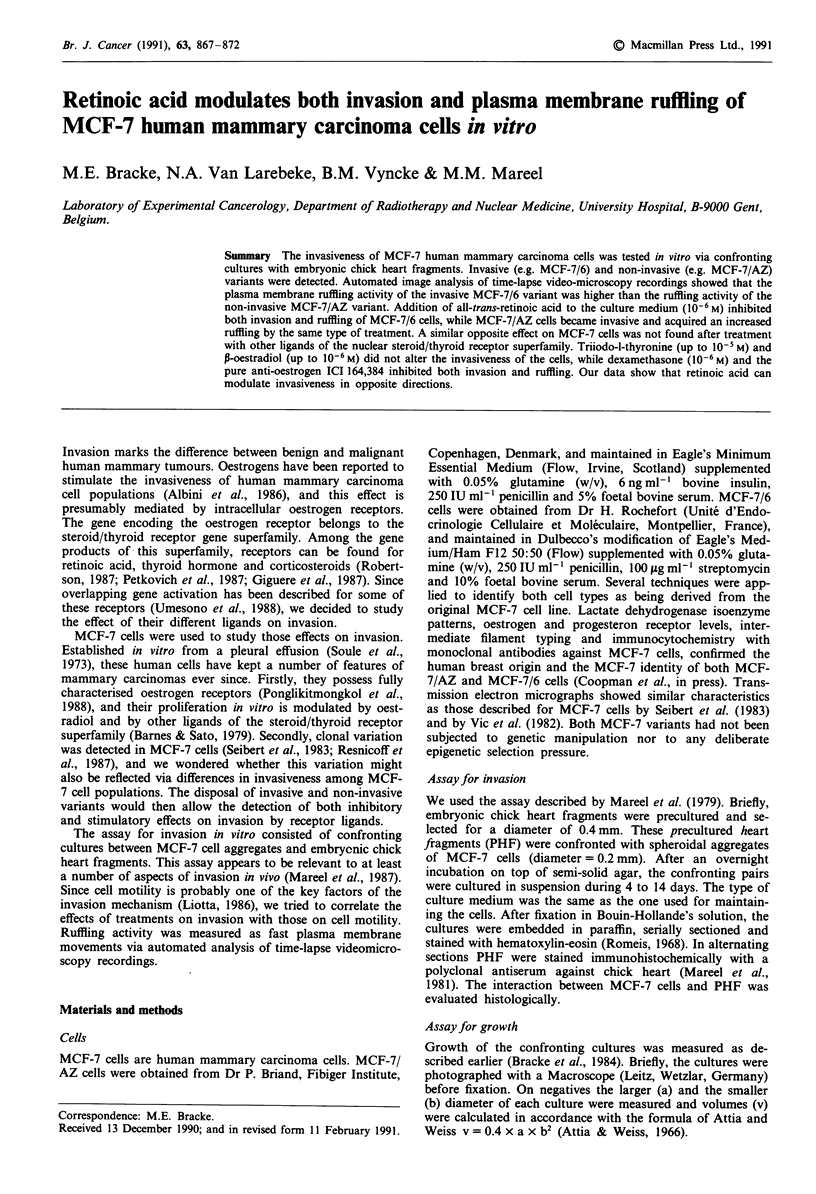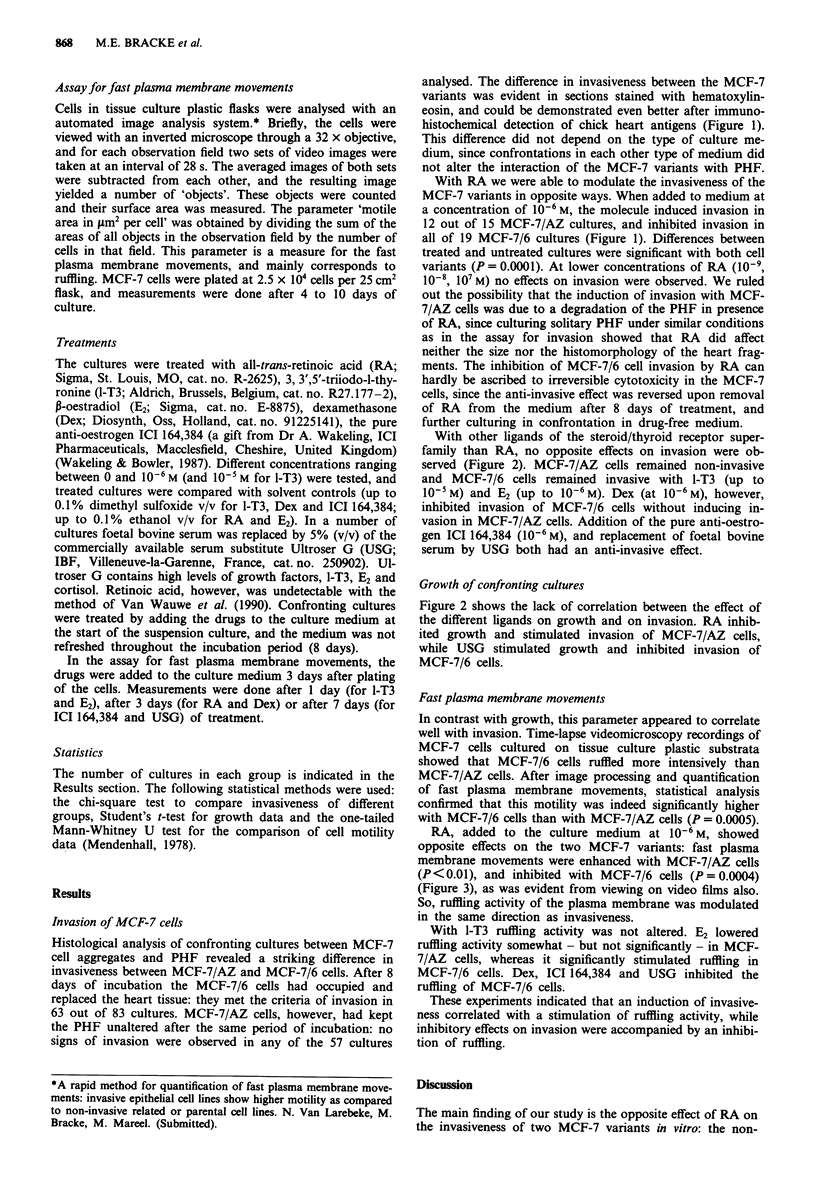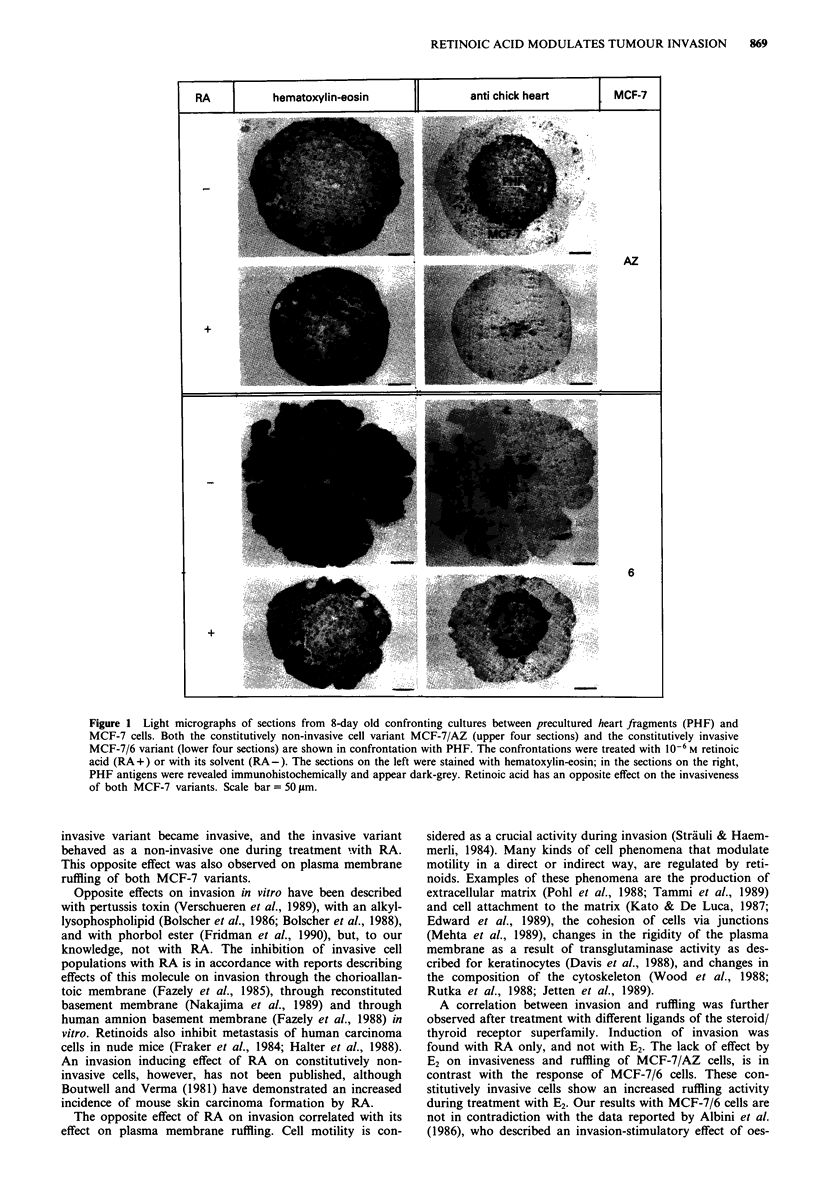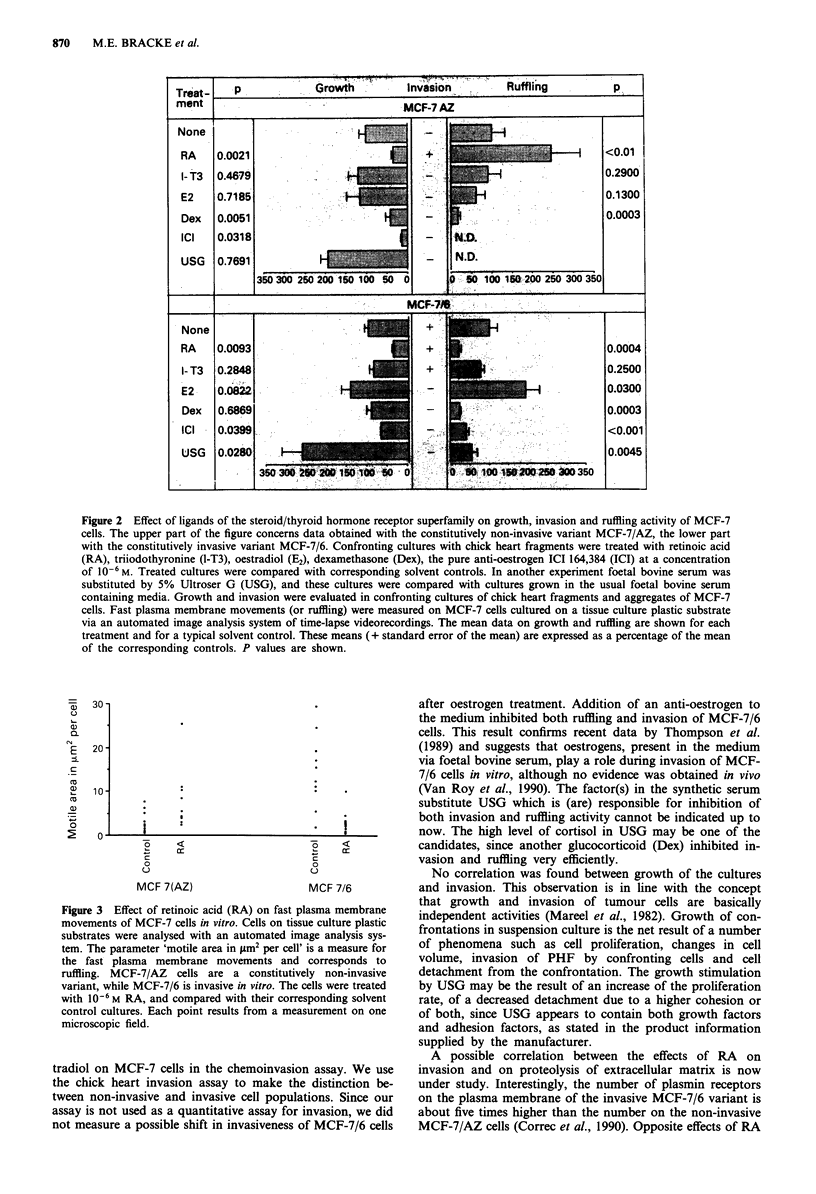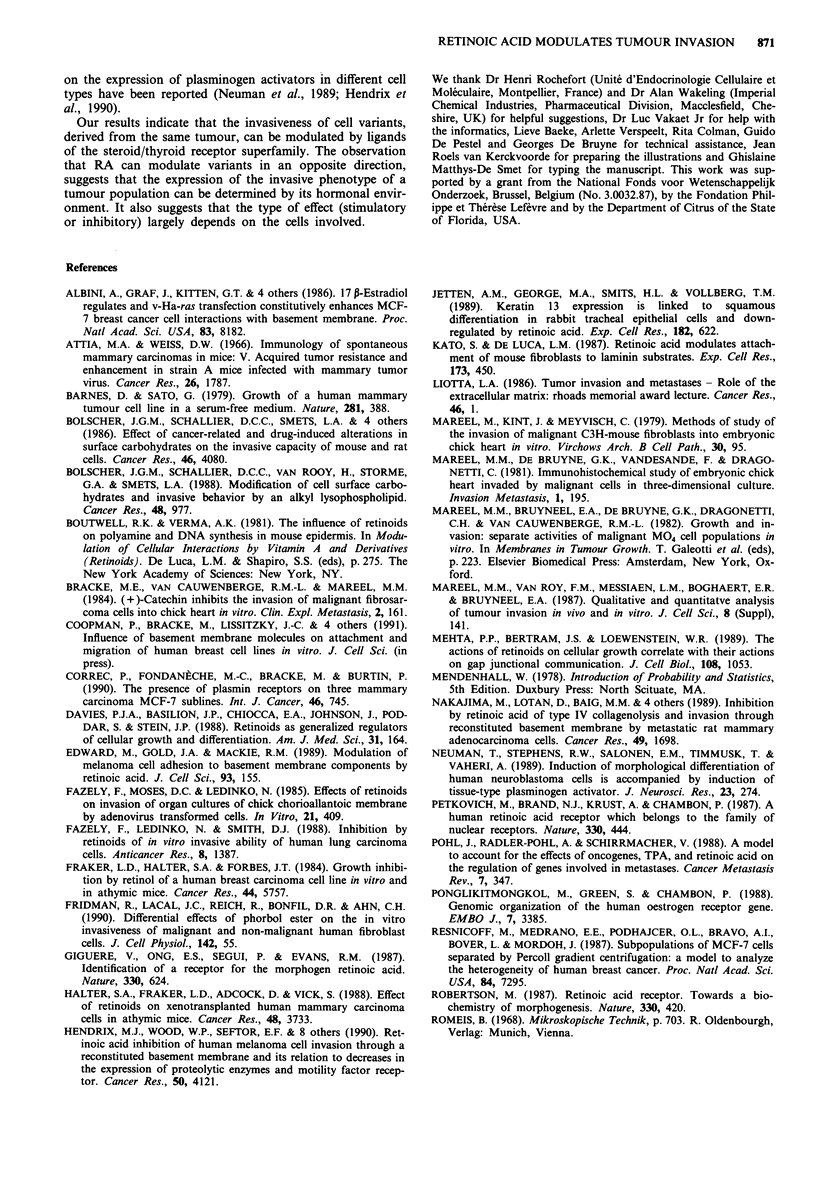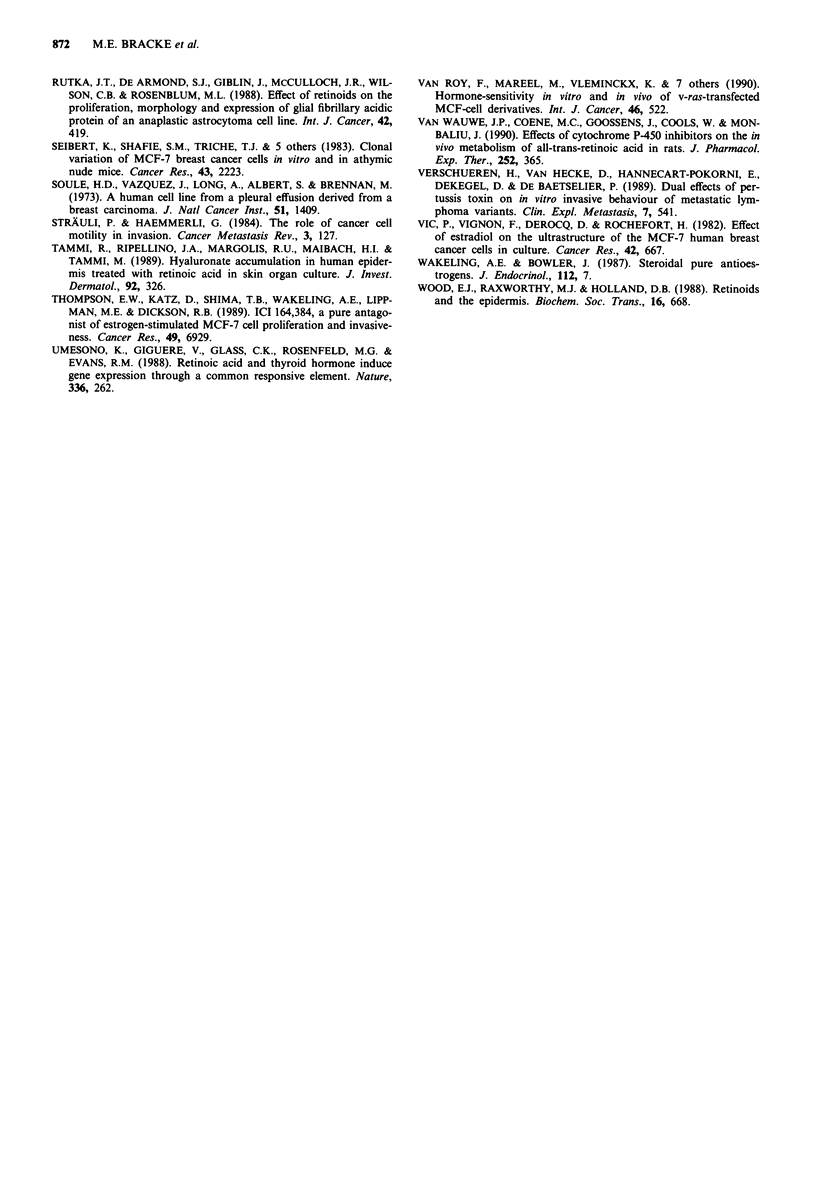# Retinoic acid modulates both invasion and plasma membrane ruffling of MCF-7 human mammary carcinoma cells in vitro.

**DOI:** 10.1038/bjc.1991.191

**Published:** 1991-06

**Authors:** M. E. Bracke, N. A. Van Larebeke, B. M. Vyncke, M. M. Mareel

**Affiliations:** Department of Radiotherapy and Nuclear Medicine, University Hospital, Gent, Belgium.

## Abstract

**Images:**


*Br. J. Cancer* (1991), **63**, 867–872 © Macmillan Press Ltd., 1991

### Retinoic acid modulates both invasion and plasma membrane ruffling of MCF-7 human mammary carcinoma cells *in vitro*

M.E. Bracke, N.A. Van Larebeke, B.M. Vyncke & M.M. Mareel

*Laboratory of Experimental Cancerology, Department of Radiotherapy and Nuclear Medicine, University Hospital, B-9000 Gent, Belgium.*

**Summary** The invasiveness of MCF-7 human mammary carcinoma cells was tested *in vitro* via confronting cultures with embryonic chick heart fragments. Invasive (e.g. MCF-7/6) and non-invasive (e.g. MCF-7/AZ) variants were detected. Automated image analysis of time-lapse video-microscopy recordings showed that the plasma membrane ruffling activity of the invasive MCF-7/6 variant was higher than the ruffling activity of the non-invasive MCF-7/AZ variant. Addition of all-*trans*-retinoic acid to the culture medium ($10^{-6}$ M) inhibited both invasion and ruffling of MCF-7/6 cells, while MCF-7/AZ cells became invasive and acquired an increased ruffling by the same type of treatment. A similar opposite effect on MCF-7 cells was not found after treatment with other ligands of the nuclear steroid/thyroid receptor superfamily. Triiodo-l-thyronine (up to $10^{-5}$ M) and $\beta$-oestradiol (up to $10^{-6}$ M) did not alter the invasiveness of the cells, while dexamethasone ($10^{-6}$ M) and the pure anti-oestrogen ICI 164,384 inhibited both invasion and ruffling. Our data show that retinoic acid can modulate invasiveness in opposite directions.

Invasion marks the difference between benign and malignant human mammary tumours. Oestrogens have been reported to stimulate the invasiveness of human mammary carcinoma cell populations (Albini *et al.*, 1986), and this effect is presumably mediated by intracellular oestrogen receptors. The gene encoding the oestrogen receptor belongs to the steroid/thyroid receptor gene superfamily. Among the gene products of this superfamily, receptors can be found for retinoic acid, thyroid hormone and corticosteroids (Robertson, 1987; Petkovich *et al.*, 1987; Giguere *et al.*, 1987). Since overlapping gene activation has been described for some of these receptors (Umesono *et al.*, 1988), we decided to study the effect of their different ligands on invasion.

MCF-7 cells were used to study those effects on invasion. Established *in vitro* from a pleural effusion (Soule *et al.*, 1973), these human cells have kept a number of features of mammary carcinomas ever since. Firstly, they possess fully characterised oestrogen receptors (Ponglikitmongkol *et al.*, 1988), and their proliferation *in vitro* is modulated by oestradiol and by other ligands of the steroid/thyroid receptor superfamily (Barnes & Sato, 1979). Secondly, clonal variation was detected in MCF-7 cells (Seibert *et al.*, 1983; Resnicoff *et al.*, 1987), and we wondered whether this variation might also be reflected via differences in invasiveness among MCF-7 cell populations. The disposal of invasive and non-invasive variants would then allow the detection of both inhibitory and stimulatory effects on invasion by receptor ligands.

The assay for invasion *in vitro* consisted of confronting cultures between MCF-7 cell aggregates and embryonic chick heart fragments. This assay appears to be relevant to at least a number of aspects of invasion *in vivo* (Mareel *et al.*, 1987). Since cell motility is probably one of the key factors of the invasion mechanism (Liotta, 1986), we tried to correlate the effects of treatments on invasion with those on cell motility. Ruffling activity was measured as fast plasma membrane movements via automated analysis of time-lapse videomicroscopy recordings.

### Materials and methods

### Cells

MCF-7 cells are human mammary carcinoma cells. MCF-7/AZ cells were obtained from Dr P. Briand, Fibiger Institute, Copenhagen, Denmark, and maintained in Eagle's Minimum Essential Medium (Flow, Irvine, Scotland) supplemented with 0.05% glutamine (w/v), 6 ng ml$^{-1}$ bovine insulin, 250 IU ml$^{-1}$ penicillin and 5% foetal bovine serum. MCF-7/6 cells were obtained from Dr H. Rochefort (Unité d'Endocrinologie Cellulaire et Moléculaire, Montpellier, France), and maintained in Dulbecco's modification of Eagle's Medium/Ham F12 50:50 (Flow) supplemented with 0.05% glutamine (w/v), 250 IU ml$^{-1}$ penicillin, 100 $\mu$g ml$^{-1}$ streptomycin and 10% foetal bovine serum. Several techniques were applied to identify both cell types as being derived from the original MCF-7 cell line. Lactate dehydrogenase isoenzyme patterns, oestrogen and progesteron receptor levels, intermediate filament typing and immunocytochemistry with monoclonal antibodies against MCF-7 cells, confirmed the human breast origin and the MCF-7 identity of both MCF-7/AZ and MCF-7/6 cells (Coopman *et al.*, in press). Transmission electron micrographs showed similar characteristics as those described for MCF-7 cells by Seibert *et al.* (1983) and by Vic *et al.* (1982). Both MCF-7 variants had not been subjected to genetic manipulation nor to any deliberate epigenetic selection pressure.

### Assay for invasion

We used the assay described by Mareel *et al.* (1979). Briefly, embryonic chick heart fragments were precultured and selected for a diameter of 0.4 mm. These *p*recultured *h*eart *f*ragments (PHF) were confronted with spheroidal aggregates of MCF-7 cells (diameter = 0.2 mm). After an overnight incubation on top of semi-solid agar, the confronting pairs were cultured in suspension during 4 to 14 days. The type of culture medium was the same as the one used for maintaining the cells. After fixation in Bouin-Hollande's solution, the cultures were embedded in paraffin, serially sectioned and stained with hematoxylin-eosin (Romeis, 1968). In alternating sections PHF were stained immunohistochemically with a polyclonal antiserum against chick heart (Mareel *et al.*, 1981). The interaction between MCF-7 cells and PHF was evaluated histologically.

### Assay for growth

Growth of the confronting cultures was measured as described earlier (Bracke *et al.*, 1984). Briefly, the cultures were photographed with a Macroscope (Leitz, Wetzlar, Germany) before fixation. On negatives the larger (a) and the smaller (b) diameter of each culture were measured and volumes (v) were calculated in accordance with the formula of Attia and Weiss $v = 0.4 \times a \times b^2$ (Attia & Weiss, 1966).

Correspondence: M.E. Bracke.
Received 13 December 1990; and in revised form 11 February 1991.

### Assay for fast plasma membrane movements

Cells in tissue culture plastic flasks were analysed with an automated image analysis system.* Briefly, the cells were viewed with an inverted microscope through a 32 × objective, and for each observation field two sets of video images were taken at an interval of 28 s. The averaged images of both sets were subtracted from each other, and the resulting image yielded a number of 'objects'. These objects were counted and their surface area was measured. The parameter 'motile area in $\mu m^2$ per cell' was obtained by dividing the sum of the areas of all objects in the observation field by the number of cells in that field. This parameter is a measure for the fast plasma membrane movements, and mainly corresponds to ruffling. MCF-7 cells were plated at $2.5 \times 10^4$ cells per 25 $cm^2$ flask, and measurements were done after 4 to 10 days of culture.

### Treatments

The cultures were treated with all-*trans*-retinoic acid (RA; Sigma, St. Louis, MO, cat. no. R-2625), 3, 3′,5′-triiodo-l-thyronine (l-T3; Aldrich, Brussels, Belgium, cat. no. R27.177–2), β-oestradiol ($E_2$; Sigma, cat. no. E-8875), dexamethasone (Dex; Diosynth, Oss, Holland, cat. no. 91225141), the pure anti-oestrogen ICI 164,384 (a gift from Dr A. Wakeling, ICI Pharmaceuticals, Macclesfield, Cheshire, United Kingdom) (Wakeling & Bowler, 1987). Different concentrations ranging between 0 and $10^{-6}$ M (and $10^{-5}$ M for l-T3) were tested, and treated cultures were compared with solvent controls (up to 0.1% dimethyl sulfoxide v/v for l-T3, Dex and ICI 164,384; up to 0.1% ethanol v/v for RA and $E_2$). In a number of cultures foetal bovine serum was replaced by 5% (v/v) of the commercially available serum substitute Ultroser G (USG; IBF, Villeneuve-la-Garenne, France, cat. no. 250902). Ultroser G contains high levels of growth factors, l-T3, $E_2$ and cortisol. Retinoic acid, however, was undetectable with the method of Van Wauwe *et al.* (1990). Confronting cultures were treated by adding the drugs to the culture medium at the start of the suspension culture, and the medium was not refreshed throughout the incubation period (8 days).

In the assay for fast plasma membrane movements, the drugs were added to the culture medium 3 days after plating of the cells. Measurements were done after 1 day (for l-T3 and $E_2$), after 3 days (for RA and Dex) or after 7 days (for ICI 164,384 and USG) of treatment.

### Statistics

The number of cultures in each group is indicated in the Results section. The following statistical methods were used: the chi-square test to compare invasiveness of different groups, Student's *t*-test for growth data and the one-tailed Mann-Whitney U test for the comparison of cell motility data (Mendenhall, 1978).

### Results

### Invasion of MCF-7 cells

Histological analysis of confronting cultures between MCF-7 cell aggregates and PHF revealed a striking difference in invasiveness between MCF-7/AZ and MCF-7/6 cells. After 8 days of incubation the MCF-7/6 cells had occupied and replaced the heart tissue: they met the criteria of invasion in 63 out of 83 cultures. MCF-7/AZ cells, however, had kept the PHF unaltered after the same period of incubation: no signs of invasion were observed in any of the 57 cultures analysed. The difference in invasiveness between the MCF-7 variants was evident in sections stained with hematoxylin-eosin, and could be demonstrated even better after immunohistochemical detection of chick heart antigens (Figure 1). This difference did not depend on the type of culture medium, since confrontations in each other type of medium did not alter the interaction of the MCF-7 variants with PHF.

With RA we were able to modulate the invasiveness of the MCF-7 variants in opposite ways. When added to medium at a concentration of $10^{-6}$ M, the molecule induced invasion in 12 out of 15 MCF-7/AZ cultures, and inhibited invasion in all of 19 MCF-7/6 cultures (Figure 1). Differences between treated and untreated cultures were significant with both cell variants ($P = 0.0001$). At lower concentrations of RA ($10^{-9}$, $10^{-8}$, $10^{7}$ M) no effects on invasion were observed. We ruled out the possibility that the induction of invasion with MCF-7/AZ cells was due to a degradation of the PHF in presence of RA, since culturing solitary PHF under similar conditions as in the assay for invasion showed that RA did affect neither the size nor the histomorphology of the heart fragments. The inhibition of MCF-7/6 cell invasion by RA can hardly be ascribed to irreversible cytotoxicity in the MCF-7 cells, since the anti-invasive effect was reversed upon removal of RA from the medium after 8 days of treatment, and further culturing in confrontation in drug-free medium.

With other ligands of the steroid/thyroid receptor superfamily than RA, no opposite effects on invasion were observed (Figure 2). MCF-7/AZ cells remained non-invasive and MCF-7/6 cells remained invasive with l-T3 (up to $10^{-5}$ M) and $E_2$ (up to $10^{-6}$ M). Dex (at $10^{-6}$ M), however, inhibited invasion of MCF-7/6 cells without inducing invasion in MCF-7/AZ cells. Addition of the pure anti-oestrogen ICI 164,384 ($10^{-6}$ M), and replacement of foetal bovine serum by USG both had an anti-invasive effect.

### Growth of confronting cultures

Figure 2 shows the lack of correlation between the effect of the different ligands on growth and on invasion. RA inhibited growth and stimulated invasion of MCF-7/AZ cells, while USG stimulated growth and inhibited invasion of MCF-7/6 cells.

### Fast plasma membrane movements

In contrast with growth, this parameter appeared to correlate well with invasion. Time-lapse videomicroscopy recordings of MCF-7 cells cultured on tissue culture plastic substrata showed that MCF-7/6 cells ruffled more intensively than MCF-7/AZ cells. After image processing and quantification of fast plasma membrane movements, statistical analysis confirmed that this motility was indeed significantly higher with MCF-7/6 cells than with MCF-7/AZ cells ($P = 0.0005$).

RA, added to the culture medium at $10^{-6}$ M, showed opposite effects on the two MCF-7 variants: fast plasma membrane movements were enhanced with MCF-7/AZ cells ($P < 0.01$), and inhibited with MCF-7/6 cells ($P = 0.0004$) (Figure 3), as was evident from viewing on video films also. So, ruffling activity of the plasma membrane was modulated in the same direction as invasiveness.

With l-T3 ruffling activity was not altered. $E_2$ lowered ruffling activity somewhat – but not significantly – in MCF-7/AZ cells, whereas it significantly stimulated ruffling in MCF-7/6 cells. Dex, ICI 164,384 and USG inhibited the ruffling of MCF-7/6 cells.

These experiments indicated that an induction of invasiveness correlated with a stimulation of ruffling activity, while inhibitory effects on invasion were accompanied by an inhibition of ruffling.

### Discussion

The main finding of our study is the opposite effect of RA on the invasiveness of two MCF-7 variants *in vitro*: the non-

*A rapid method for quantification of fast plasma membrane movements: invasive epithelial cell lines show higher motility as compared to non-invasive related or parental cell lines. N. Van Larebeke, M. Bracke, M. Mareel. (Submitted).

**Figure 1** Light micrographs of sections from 8-day old confronting cultures between *p*recultured *h*eart *f*ragments (PHF) and MCF-7 cells. Both the constitutively non-invasive cell variant MCF-7/AZ (upper four sections) and the constitutively invasive MCF-7/6 variant (lower four sections) are shown in confrontation with PHF. The confrontations were treated with $10^{-6}$ M retinoic acid (RA+) or with its solvent (RA−). The sections on the left were stained with hematoxylin-eosin; in the sections on the right, PHF antigens were revealed immunohistochemically and appear dark-grey. Retinoic acid has an opposite effect on the invasiveness of both MCF-7 variants. Scale bar = 50 μm.

invasive variant became invasive, and the invasive variant behaved as a non-invasive one during treatment with RA. This opposite effect was also observed on plasma membrane ruffling of both MCF-7 variants.

Opposite effects on invasion *in vitro* have been described with pertussis toxin (Verschueren *et al.*, 1989), with an alkyllysophospholipid (Bolscher *et al.*, 1986; Bolscher *et al.*, 1988), and with phorbol ester (Fridman *et al.*, 1990), but, to our knowledge, not with RA. The inhibition of invasive cell populations with RA is in accordance with reports describing effects of this molecule on invasion through the chorioallantoic membrane (Fazely *et al.*, 1985), through reconstituted basement membrane (Nakajima *et al.*, 1989) and through human amnion basement membrane (Fazely *et al.*, 1988) *in vitro*. Retinoids also inhibit metastasis of human carcinoma cells in nude mice (Fraker *et al.*, 1984; Halter *et al.*, 1988). An invasion inducing effect of RA on constitutively non-invasive cells, however, has not been published, although Boutwell and Verma (1981) have demonstrated an increased incidence of mouse skin carcinoma formation by RA.

The opposite effect of RA on invasion correlated with its effect on plasma membrane ruffling. Cell motility is considered as a crucial activity during invasion (Sträuli & Haemmerli, 1984). Many kinds of cell phenomena that modulate motility in a direct or indirect way, are regulated by retinoids. Examples of these phenomena are the production of extracellular matrix (Pohl *et al.*, 1988; Tammi *et al.*, 1989) and cell attachment to the matrix (Kato & De Luca, 1987; Edward *et al.*, 1989), the cohesion of cells via junctions (Mehta *et al.*, 1989), changes in the rigidity of the plasma membrane as a result of transglutaminase activity as described for keratinocytes (Davis *et al.*, 1988), and changes in the composition of the cytoskeleton (Wood *et al.*, 1988; Rutka *et al.*, 1988; Jetten *et al.*, 1989).

A correlation between invasion and ruffling was further observed after treatment with different ligands of the steroid/thyroid receptor superfamily. Induction of invasion was found with RA only, and not with $E_2$. The lack of effect by $E_2$ on invasiveness and ruffling of MCF-7/AZ cells, is in contrast with the response of MCF-7/6 cells. These constitutively invasive cells show an increased ruffling activity during treatment with $E_2$. Our results with MCF-7/6 cells are not in contradiction with the data reported by Albini *et al.* (1986), who described an invasion-stimulatory effect of oes-

**Figure 2** Effect of ligands of the steroid/thyroid hormone receptor superfamily on growth, invasion and ruffling activity of MCF-7 cells. The upper part of the figure concerns data obtained with the constitutively non-invasive variant MCF-7/AZ, the lower part with the constitutively invasive variant MCF-7/6. Confronting cultures with chick heart fragments were treated with retinoic acid (RA), triiodothyronine (l-T3), oestradiol ($E_2$), dexamethasone (Dex), the pure anti-oestrogen ICI 164,384 (ICI) at a concentration of $10^{-6}$ M. Treated cultures were compared with corresponding solvent controls. In another experiment foetal bovine serum was substituted by 5% Ultroser G (USG), and these cultures were compared with cultures grown in the usual foetal bovine serum containing media. Growth and invasion were evaluated in confronting cultures of chick heart fragments and aggregates of MCF-7 cells. Fast plasma membrane movements (or ruffling) were measured on MCF-7 cells cultured on a tissue culture plastic substrate via an automated image analysis system of time-lapse videorecordings. The mean data on growth and ruffling are shown for each treatment and for a typical solvent control. These means (+ standard error of the mean) are expressed as a percentage of the mean of the corresponding controls. *P* values are shown.

**Figure 3** Effect of retinoic acid (RA) on fast plasma membrane movements of MCF-7 cells *in vitro*. Cells on tissue culture plastic substrates were analysed with an automated image analysis system. The parameter 'motile area in $\mu m^2$ per cell' is a measure for the fast plasma membrane movements and corresponds to ruffling. MCF-7/AZ cells are a constitutively non-invasive variant, while MCF-7/6 is invasive *in vitro*. The cells were treated with $10^{-6}$ M RA, and compared with their corresponding solvent control cultures. Each point results from a measurement on one microscopic field.

tradiol on MCF-7 cells in the chemoinvasion assay. We use the chick heart invasion assay to make the distinction between non-invasive and invasive cell populations. Since our assay is not used as a quantitative assay for invasion, we did not measure a possible shift in invasiveness of MCF-7/6 cells after oestrogen treatment. Addition of an anti-oestrogen to the medium inhibited both ruffling and invasion of MCF-7/6 cells. This result confirms recent data by Thompson *et al.* (1989) and suggests that oestrogens, present in the medium via foetal bovine serum, play a role during invasion of MCF-7/6 cells *in vitro*, although no evidence was obtained *in vivo* (Van Roy *et al.*, 1990). The factor(s) in the synthetic serum substitute USG which is (are) responsible for inhibition of both invasion and ruffling activity cannot be indicated up to now. The high level of cortisol in USG may be one of the candidates, since another glucocorticoid (Dex) inhibited invasion and ruffling very efficiently.

No correlation was found between growth of the cultures and invasion. This observation is in line with the concept that growth and invasion of tumour cells are basically independent activities (Mareel *et al.*, 1982). Growth of confrontations in suspension culture is the net result of a number of phenomena such as cell proliferation, changes in cell volume, invasion of PHF by confronting cells and cell detachment from the confrontation. The growth stimulation by USG may be the result of an increase of the proliferation rate, of a decreased detachment due to a higher cohesion or of both, since USG appears to contain both growth factors and adhesion factors, as stated in the product information supplied by the manufacturer.

A possible correlation between the effects of RA on invasion and on proteolysis of extracellular matrix is now under study. Interestingly, the number of plasmin receptors on the plasma membrane of the invasive MCF-7/6 variant is about five times higher than the number on the non-invasive MCF-7/AZ cells (Correc *et al.*, 1990). Opposite effects of RA

on the expression of plasminogen activators in different cell types have been reported (Neuman *et al.*, 1989; Hendrix *et al.*, 1990).

Our results indicate that the invasiveness of cell variants, derived from the same tumour, can be modulated by ligands of the steroid/thyroid receptor superfamily. The observation that RA can modulate variants in an opposite direction, suggests that the expression of the invasive phenotype of a tumour population can be determined by its hormonal environment. It also suggests that the type of effect (stimulatory or inhibitory) largely depends on the cells involved.

We thank Dr Henri Rochefort (Unité d'Endocrinologie Cellulaire et Moléculaire, Montpellier, France) and Dr Alan Wakeling (Imperial Chemical Industries, Pharmaceutical Division, Macclesfield, Cheshire, UK) for helpful suggestions, Dr Luc Vakaet Jr for help with the informatics, Lieve Baeke, Arlette Verspeelt, Rita Colman, Guido De Pestel and Georges De Bruyne for technical assistance, Jean Roels van Kerckvoorde for preparing the illustrations and Ghislaine Matthys-De Smet for typing the manuscript. This work was supported by a grant from the National Fonds voor Wetenschappelijk Onderzoek, Brussel, Belgium (No. 3.0032.87), by the Fondation Philippe et Thérèse Lefèvre and by the Department of Citrus of the State of Florida, USA.

### References

ALBINI, A., GRAF, J., KITTEN, G.T. & 4 others (1986). 17 β-Estradiol regulates and v-Ha-*ras* transfection constitutively enhances MCF-7 breast cancer cell interactions with basement membrane. *Proc. Natl Acad. Sci. USA*, **83**, 8182.

ATTIA, M.A. & WEISS, D.W. (1966). Immunology of spontaneous mammary carcinomas in mice: V. Acquired tumor resistance and enhancement in strain A mice infected with mammary tumor virus. *Cancer Res.*, **26**, 1787.

BARNES, D. & SATO, G. (1979). Growth of a human mammary tumour cell line in a serum-free medium. *Nature*, **281**, 388.

BOLSCHER, J.G.M., SCHALLIER, D.C.C., SMETS, L.A. & 4 others (1986). Effect of cancer-related and drug-induced alterations in surface carbohydrates on the invasive capacity of mouse and rat cells. *Cancer Res.*, **46**, 4080.

BOLSCHER, J.G.M., SCHALLIER, D.C.C., VAN ROOY, H., STORME, G.A. & SMETS, L.A. (1988). Modification of cell surface carbohydrates and invasive behavior by an alkyl lysophospholipid. *Cancer Res.*, **48**, 977.

BOUTWELL, R.K. & VERMA, A.K. (1981). The influence of retinoids on polyamine and DNA synthesis in mouse epidermis. In *Modulation of Cellular Interactions by Vitamin A and Derivatives (Retinoids)*. De Luca, L.M. & Shapiro, S.S. (eds), p. 275. The New York Academy of Sciences: New York, NY.

BRACKE, M.E., VAN CAUWENBERGE, R.M.-L. & MAREEL, M.M. (1984). (+)-Catechin inhibits the invasion of malignant fibrosarcoma cells into chick heart *in vitro*. *Clin. Expl. Metastasis*, **2**, 161.

COOPMAN, P., BRACKE, M., LISSITZKY, J.-C. & 4 others (1991). Influence of basement membrane molecules on attachment and migration of human breast cell lines *in vitro*. *J. Cell Sci.* (in press).

CORREC, P., FONDANÈCHE, M.-C., BRACKE, M. & BURTIN, P. (1990). The presence of plasmin receptors on three mammary carcinoma MCF-7 sublines. *Int. J. Cancer*, **46**, 745.

DAVIES, P.J.A., BASILION, J.P., CHIOCCA, E.A., JOHNSON, J., PODDAR, S. & STEIN, J.P. (1988). Retinoids as generalized regulators of cellular growth and differentiation. *Am. J. Med. Sci.*, **31**, 164.

EDWARD, M., GOLD, J.A. & MACKIE, R.M. (1989). Modulation of melanoma cell adhesion to basement membrane components by retinoic acid. *J. Cell Sci.*, **93**, 155.

FAZELY, F., MOSES, D.C. & LEDINKO, N. (1985). Effects of retinoids on invasion of organ cultures of chick chorioallantoic membrane by adenovirus transformed cells. *In Vitro*, **21**, 409.

FAZELY, F., LEDINKO, N. & SMITH, D.J. (1988). Inhibition by retinoids of *in vitro* invasive ability of human lung carcinoma cells. *Anticancer Res.*, **8**, 1387.

FRAKER, L.D., HALTER, S.A. & FORBES, J.T. (1984). Growth inhibition by retinol of a human breast carcinoma cell line *in vitro* and in athymic mice. *Cancer Res.*, **44**, 5757.

FRIDMAN, R., LACAL, J.C., REICH, R., BONFIL, D.R. & AHN, C.H. (1990). Differential effects of phorbol ester on the in vitro invasiveness of malignant and non-malignant human fibroblast cells. *J. Cell Physiol.*, **142**, 55.

GIGUERE, V., ONG, E.S., SEGUI, P. & EVANS, R.M. (1987). Identification of a receptor for the morphogen retinoic acid. *Nature*, **330**, 624.

HALTER, S.A., FRAKER, L.D., ADCOCK, D. & VICK, S. (1988). Effect of retinoids on xenotransplanted human mammary carcinoma cells in athymic mice. *Cancer Res.*, **48**, 3733.

HENDRIX, M.J., WOOD, W.P., SEFTOR, E.F. & 8 others (1990). Retinoic acid inhibition of human melanoma cell invasion through a reconstituted basement membrane and its relation to decreases in the expression of proteolytic enzymes and motility factor receptor. *Cancer Res.*, **50**, 4121.

JETTEN, A.M., GEORGE, M.A., SMITS, H.L. & VOLLBERG, T.M. (1989). Keratin 13 expression is linked to squamous differentiation in rabbit tracheal epithelial cells and down-regulated by retinoic acid. *Exp. Cell Res.*, **182**, 622.

KATO, S. & DE LUCA, L.M. (1987). Retinoic acid modulates attachment of mouse fibroblasts to laminin substrates. *Exp. Cell Res.*, **173**, 450.

LIOTTA, L.A. (1986). Tumor invasion and metastases – Role of the extracellular matrix: rhoads memorial award lecture. *Cancer Res.*, **46**, 1.

MAREEL, M., KINT, J. & MEYVISCH, C. (1979). Methods of study of the invasion of malignant C3H-mouse fibroblasts into embryonic chick heart *in vitro*. *Virchows Arch. B Cell Path.*, **30**, 95.

MAREEL, M.M., DE BRUYNE, G.K., VANDESANDE, F. & DRAGONETTI, C. (1981). Immunohistochemical study of embryonic chick heart invaded by malignant cells in three-dimensional culture. *Invasion Metastasis*, **1**, 195.

MAREEL, M.M., BRUYNEEL, E.A., DE BRUYNE, G.K., DRAGONETTI, C.H. & VAN CAUWENBERGE, R.M.-L. (1982). Growth and invasion: separate activities of malignant $MO_4$ cell populations *in vitro*. In *Membranes in Tumour Growth*. T. Galeotti *et al.* (eds), p. 223. Elsevier Biomedical Press: Amsterdam, New York, Oxford.

MAREEL, M.M., VAN ROY, F.M., MESSIAEN, L.M., BOGHAERT, E.R. & BRUYNEEL, E.A. (1987). Qualitative and quantitatve analysis of tumour invasion *in vivo* and *in vitro*. *J. Cell Sci.*, **8** (Suppl), 141.

MEHTA, P.P., BERTRAM, J.S. & LOEWENSTEIN, W.R. (1989). The actions of retinoids on cellular growth correlate with their actions on gap junctional communication. *J. Cell Biol.*, **108**, 1053.

MENDENHALL, W. (1978). *Introduction of Probability and Statistics*, 5th Edition. Duxbury Press: North Scituate, MA.

NAKAJIMA, M., LOTAN, D., BAIG, M.M. & 4 others (1989). Inhibition by retinoic acid of type IV collagenolysis and invasion through reconstituted basement membrane by metastatic rat mammary adenocarcinoma cells. *Cancer Res.*, **49**, 1698.

NEUMAN, T., STEPHENS, R.W., SALONEN, E.M., TIMMUSK, T. & VAHERI, A. (1989). Induction of morphological differentiation of human neuroblastoma cells is accompanied by induction of tissue-type plasminogen activator. *J. Neurosci. Res.*, **23**, 274.

PETKOVICH, M., BRAND, N.J., KRUST, A. & CHAMBON, P. (1987). A human retinoic acid receptor which belongs to the family of nuclear receptors. *Nature*, **330**, 444.

POHL, J., RADLER-POHL, A. & SCHIRRMACHER, V. (1988). A model to account for the effects of oncogenes, TPA, and retinoic acid on the regulation of genes involved in metastases. *Cancer Metastasis Rev.*, **7**, 347.

PONGLIKITMONGKOL, M., GREEN, S. & CHAMBON, P. (1988). Genomic organization of the human oestrogen receptor gene. *EMBO J.*, **7**, 3385.

RESNICOFF, M., MEDRANO, E.E., PODHAJCER, O.L., BRAVO, A.I., BOVER, L. & MORDOH, J. (1987). Subpopulations of MCF-7 cells separated by Percoll gradient centrifugation: a model to analyze the heterogeneity of human breast cancer. *Proc. Natl Acad. Sci. USA*, **84**, 7295.

ROBERTSON, M. (1987). Retinoic acid receptor. Towards a biochemistry of morphogenesis. *Nature*, **330**, 420.

ROMEIS, B. (1968). *Mikroskopische Technik*, p. 703. R. Oldenbourgh, Verlag: Munich, Vienna.

RUTKA, J.T., DE ARMOND, S.J., GIBLIN, J., MCCULLOCH, J.R., WILSON, C.B. & ROSENBLUM, M.L. (1988). Effect of retinoids on the proliferation, morphology and expression of glial fibrillary acidic protein of an anaplastic astrocytoma cell line. *Int. J. Cancer*, **42**, 419.

SEIBERT, K., SHAFIE, S.M., TRICHE, T.J. & 5 others (1983). Clonal variation of MCF-7 breast cancer cells *in vitro* and in athymic nude mice. *Cancer Res.*, **43**, 2223.

SOULE, H.D., VAZQUEZ, J., LONG, A., ALBERT, S. & BRENNAN, M. (1973). A human cell line from a pleural effusion derived from a breast carcinoma. *J. Natl Cancer Inst.*, **51**, 1409.

STRÄULI, P. & HAEMMERLI, G. (1984). The role of cancer cell motility in invasion. *Cancer Metastasis Rev.*, **3**, 127.

TAMMI, R., RIPELLINO, J.A., MARGOLIS, R.U., MAIBACH, H.I. & TAMMI, M. (1989). Hyaluronate accumulation in human epidermis treated with retinoic acid in skin organ culture. *J. Invest. Dermatol.*, **92**, 326.

THOMPSON, E.W., KATZ, D., SHIMA, T.B., WAKELING, A.E., LIPPMAN, M.E. & DICKSON, R.B. (1989). ICI 164,384, a pure antagonist of estrogen-stimulated MCF-7 cell proliferation and invasiveness. *Cancer Res.*, **49**, 6929.

UMESONO, K., GIGUERE, V., GLASS, C.K., ROSENFELD, M.G. & EVANS, R.M. (1988). Retinoic acid and thyroid hormone induce gene expression through a common responsive element. *Nature*, **336**, 262.

VAN ROY, F., MAREEL, M., VLEMINCKX, K. & 7 others (1990). Hormone-sensitivity *in vitro* and *in vivo* of v-*ras*-transfected MCF-cell derivatives. *Int. J. Cancer*, **46**, 522.

VAN WAUWE, J.P., COENE, M.C., GOOSSENS, J., COOLS, W. & MONBALIU, J. (1990). Effects of cytochrome P-450 inhibitors on the *in vivo* metabolism of all-trans-retinoic acid in rats. *J. Pharmacol. Exp. Ther.*, **252**, 365.

VERSCHUEREN, H., VAN HECKE, D., HANNECART-POKORNI, E., DEKEGEL, D. & DE BAETSELIER, P. (1989). Dual effects of pertussis toxin on *in vitro* invasive behaviour of metastatic lymphoma variants. *Clin. Expl. Metastasis*, **7**, 541.

VIC, P., VIGNON, F., DEROCQ, D. & ROCHEFORT, H. (1982). Effect of estradiol on the ultrastructure of the MCF-7 human breast cancer cells in culture. *Cancer Res.*, **42**, 667.

WAKELING, A.E. & BOWLER, J. (1987). Steroidal pure antioestrogens. *J. Endocrinol.*, **112**, 7.

WOOD, E.J., RAXWORTHY, M.J. & HOLLAND, D.B. (1988). Retinoids and the epidermis. *Biochem. Soc. Trans.*, **16**, 668.